# *HLA-DQB1*/*DRB1* Alleles Associate with Traditional Chinese Medicine Syndrome of Chronic Hepatitis B: A Potential Predictor of Progression

**DOI:** 10.1155/2019/8146937

**Published:** 2019-11-21

**Authors:** Xiyang Liu, Tingjun Wan, Sijie Dang, Dong Wang, Cen Jiang, Yue Su, Mengmeng Shen, Xuchen Tang, Xia Li, Baixue Li, Li Wen, Quansheng Feng

**Affiliations:** ^1^Chengdu University of Traditional Chinese Medicine, Chengdu 610075, China; ^2^Sichuan Second Hospital of Traditional Chinese Medicine, Chengdu 610041, China; ^3^Leshan Vocational and Technical College, Leshan 614000, China; ^4^Pengzhou Hospital of Traditional Chinese Medicine, Pengzhou 611930, China

## Abstract

**Background and Aims:**

Traditional Chinese medicine (TCM) has been widely applied in chronic hepatitis B (CHB) supplementary treatment in China. Kidney yang deficiency syndrome (KYDS), one of the most common TCM syndromes of CHB, is more likely to progress to liver cirrhosis or hepatocellular carcinoma than other syndromes. Polymorphisms in the human leucocyte antigen- (HLA-) *DQB1* and *-DRB1* genes were reported to be associated with hepatitis B virus infection outcomes. Here, we investigated whether *HLA-DQB1* and *HLA-DRB1* are associated with the classification of CHB TCM syndromes.

**Methods:**

We genotyped *HLA-DQB1* and *HLA-DRB1* alleles in a total of 105 subjects, including 74 CHB patients (28 KYDS and 46 non-KYDS) and 31 healthy individuals from Sichuan Province of Southwest China, by polymerase chain reaction sequence-based typing (PCR-SBT). Moreover, a meta-analysis was carried out for further verification.

**Results:**

The proportion of patients with high HBV DNA load (≥2000 IU/ml) in the KYDS group is higher than that in the non-KYDS group (60.70% [17/28] vs. 28.30% [13/46]); *P*=0.01). The frequencies of *HLA-DQB1∗02*:*01* (*P*=0.04) and *HLA-DRB1∗03*:*01* (*P*=0.04) in the KYDS group were significantly increased compared to the non-KYDS group. The gene test and meta-analysis showed that *HLA-DRB1∗08*:*03* confers susceptibility to CHB (odds ratio = 1.57).

**Conclusion:**

We found an association between *HLA-DRB1*/*DQB1* polymorphisms and KYDS of CHB. Moreover, KYDS patients of CHB are characteristic with high HBV DNA loads. These findings help to reveal the biological mechanism of KYDS in high risk of CHB progression and suggest a potential prognostic value for disease outcome evaluation.

## 1. Introduction

Although lots of strategies have been applied [1], hepatitis B virus (HBV) infection is still a worldwide public health threat that causes considerable liver-related morbidity and mortality. Indeed, 257 million people worldwide are living with HBV infection and 887000 dead from complications (including liver cirrhosis and hepatocellular carcinoma) [2]. Low screening and diagnosis rate of HBV infection, resulting in three-quarters of men even did not know they have been infected [3]. Therefore, in addition to liver biochemistry, virological markers, and abdominal ultrasonography, a noninvasive and convenient assessment of disease progression is emerging as an important assessment modality [4].

Traditional Chinese medicine (TCM) is a systematic medical method with a history of more than 2,000 years in China. A nationwide population-based cohort study showed that Chinese herbal medicine could reduce HCC risk in patients with CHB [5]. Compared with the treatment of nucleoside analogues (NAs) or interferons (IFNs), TCM therapies have equivalent or better effect in HBeAg and HBsAg seroconversion as well as HBV DNA clearance [6, 7]. TCM syndrome is the key to TCM diagnosis and treatment. Kidney yang deficiency syndrome (KYDS) is one of the most common TCM syndromes in patients with severe CHB [8] and the most common type in chronic HBV carriers in immunotolerant phase [9]. It has been reported that patients of KYDS with CHB had a higher risk to progress to LC or HCC than the other syndromes [10]. Besides, KYDS was reported to be closely related to hypoimmunity and weakness of antioxidation capacity [11]. CHB patients of KYDS possess lower levels of CD4+ T cells, IL-2, and IFN-*γ* than the other TCM syndromes, while the levels of IL-6 and IL-10 in KYDS were higher [12]. However, it lacks biological evidence for the clinical and epidemiological features of KYDS of CHB.

Genome-wide association studies (GWASs) have identified several human leukocyte antigen (HLA) class II genes associated with outcomes of HBV infection, response to HBV vaccines, and antiviral efficacy of IFN and NAs [13–16]. HLA class II proteins are expressed in antigen presenting cells. It plays a critical role in the immune system by presenting peptides derived from extracellular protein [17]. *HLA-DQB1* and *HLA-DRB1* belong to HLA class II beta chain paralogs. According to high-throughput sequencing (HTS), most gene variants located in *HLA-BQB1* and *HLA-DRB1* were found to be associated with CHB [18]. Based on the prominent autoimmune features in KYDS, we hypothesis there should be a close association between HLA-DQB1/DRB1 and KYDS.

In this study, we aimed to examine the possible association of genetic variants of *HLA-DQB1*/*DRB1* and patients of KDYS with CHB in Han Chinese cohort. Our results would provide evidence that TCM syndromes were related to genetic background.

## 2. Materials and Methods

### 2.1. Patients

A total of 74 CHB patients (28 KYDS and 46 non-KYDS) were enrolled in the Affiliated Hospital of TCM of Southwest Medical University, Chengdu Public Health Clinical Medical Center, and Pengzhou Hospital of TCM from Sichuan Province in Southwest China. All the enrolled patients should be diagnosed as CHB according to *the guideline of prevention and treatment for chronic hepatitis B (2015 version)* [19]: with positive HBsAg for more than six months, with persistent or intermittent elevation in ALT/AST levels or liver biopsy showing chronic hepatitis, and with positive HBV DNA viral load. The diagnostic criteria of KYDS refer to *Diagnostics of Traditional Chinese Medicine (2002 edition)* [20] (Supplementary [Supplementary-material supplementary-material-1]). The non-KYDS patients were those who diagnosed with the other type of TCM syndromes. All the patients should be satisfied with the inclusion and exclusion criteria (Supplementary [Supplementary-material supplementary-material-1]). Thirty-one individuals with healthy physical examination were enrolled in the control group. All the participants in this study were Han Chinese and provided written informed consent for participation. This study was approved by the research medical ethics committee of Chengdu University of TCM (China).

### 2.2. Serological and Biochemical Tests

Each blood sample (4 ml) was collected from included participants and stored at −80°C preparing for testing. HBsAg, anti-HBs, HBeAg, anti-HBe, and anti-HBc were screened by enzyme-linked immunosorbent assay (Roche, Switzerland). HBV DNA was quantitated by fluorescence quantitative PCR (Roche, Switzerland).

### 2.3. DNA Extraction and HLA-DQB1/HLA-DRB1 Gene Genotyping

Genomic DNA was extracted from whole blood using the QIAamp DNA Blood mini kit (Qiagen, Hilden, Germany). The extracted DNA should be satisfied with concentration of 20–100 ng/*µ*l, purity (260/280) of 1.65–1.95, and minimum volume of 15 *µ*l. The prepared samples should be stored at 2–8°C and tested within 48 hours, otherwise frozen at −20°C or below. *HLA-DQB1* and *-DRB1* were genotyped by polymerase chain reaction sequence-based typing (PCR-SBT). Primers were designed based on references [21–23]. The PCR amplification conditions were as follows: initial denaturation at 95°C for 2 minutes, denaturation at 94°C for 20 seconds, annealing at 55°C for 50 seconds, extension at 72°C for 30 seconds, with a total of 30 cycles, and finally, extension at 72°C for 5 minutes.

### 2.4. Meta-Analysis Study

In order to verify the results of genetic research by PCR-SBT, we applied a meta-analysis. PubMed, Web of Science, and China National Knowledge Internet (CNKI) databases were used to search studies which reported the association of *HLA-DRB1∗08* with CHB, using subject heading terms “hepatitis B virus” or “chronic hepatitis B” and “*HLA-DRB1*” or “*HLA-DRB1∗08.*” The inclusion and exclusion criteria are shown in Supplementary [Supplementary-material supplementary-material-1]. For avoiding the racial influence, all the regions of the researches were set in China.

### 2.5. Statistical Analysis

Clinical data were shown as means ± SD or median and interquartile range. *T*-test or Mann–Whitney *U* test was used to compare groups. Allele frequencies were compared between the CHB group and healthy control group, as well as the KYDS and non-KYDS group. The chi-squared test or two-tailed Fisher's exact test were applied in the comparison. All analyses were performed using SPSS 20.0 (SPSS Inc., Chicago, Illinois). The Hardy–Weinberg equilibrium test was examined by the Arlequin software version 3.5 (http://anthro.unige.ch/arlequin). Meta-analysis was performed by software Revman 5.3 (http://tech.cochrane.org/revman/download). The random effect model was used when there was a heterogeneity (*I*^2^ > 50%), and a fixed effect model was used if *I*^2^ < 50%. The Newcastle–Ottawa Scale (NOS) (http://www.ohri.ca/programs/clinical_epidemiology/oxford.asp) was used to evaluate the quality of this study. *P* < 0.05 was considered statistically significant.

## 3. Results

### 3.1. Patients Characteristics

There was no significant difference in age and gender between the KYDS group, non-KYDS group and healthy control group (*P* > 0.05). Serum aspartate aminotransferase (AST) levels, alanine aminotransferase (ALT) levels, total bilirubin (TBIL) levels, and serological HBV markers had no significant difference between the KYDS group and non-KYDS group (*P* > 0.05) (Table 1). However, patients with high HBV DNA load (≥2000 IU/ml) in the KYDS group showed a significant increase than non-KYDS group patients, accounting for 60.70% (17/28) and 28.30% (13/46), respectively (*P*=0.01, OR = 3.53, 95% CI 1.32–9.46).

### 3.2. Distribution of HLA-DQB1 and HLA-DRB1 Alleles in Subjects

There was no significant deviation from Hardy–Weinberg equilibrium, with *P* value >0.05 (supporting [Supplementary-material supplementary-material-1]). The frequency of *HLA-DQB1∗03*:*01* showed a significant difference between the CHB group and control group. The frequency of subjects carrying *HLA-DQB1∗03*:*01* was 30.65% among the control group more than 19.59% among the CHB group (*P*=0.04, OR = 0.41, 95% CI: 0.17–0.96). The frequency of *HLA-DRB1∗12*:*01* showed a significant difference between the CHB group and HC group (1.35% versus 8.06%, *P*=0.02, OR = 0.14, 95% CI: 0.03–0.79). Additionally, the frequency of *HLA-DRB1∗08*:*03* in the CHB group was significantly increased compared with the HC group (10.14% versus 1.61%, *P*=0.04, OR = 7.63, 95% CI: 0.96–60.53) (Table 2).

The frequency of *HLA-DQB1∗02*:*01* was significantly increased in the KYDS group than in the non-KYDS group (14.29% versus 4.35%, *P*=0.04, OR = 4.20, 95% CI: 1.13–15.61). The frequency of subjects carrying *HLA-DRB1∗03*:*01* was 14.29% among the KYDS group, whereas it was only 4.35% among the non-KYDS group (*P*=0.04, OR = 4.20, 95% CI: 1.13–15.61) (Table 3).

### 3.3. *HLA-DRB1∗08:03* Associated with CHB in Meta-Analysis

**Figure 1:**
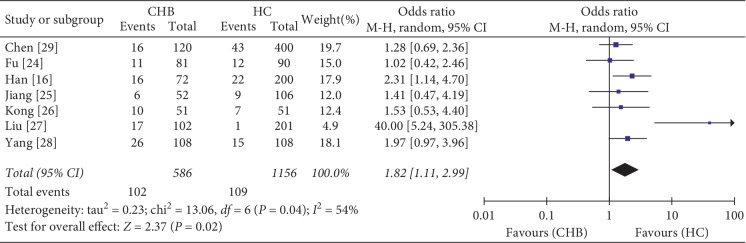
Forest plots of pooled odds ratio with 95% confidence interval for associations between *HLA-DRB1∗08* and CHB. CHB, patients with chronic hepatitis B infection; HC, health control; OR, odds ratio; CI, confidence interval.

According to the clinical research, *HLA-DRB1∗08*:*03* was a susceptible allele to CHB infection. Meta-analysis was used to further validate the result. In total, 69 potential studies were identified and screened until March 19, 2019. Finally, 1742 subjects (586 cases and 1156 controls) from seven case-control cohorts were included in this meta-analysis. Characteristics of these studies are shown in Table 4. The scores of NOS of seven studies showed that the median score was 6, indicating a high methodological quality. The results indicated that *HLA-DRB1∗08* had a significant association with CHB and was a risk gene for this disease (*I*^2^ = 54%, *P*=0.02, OR = 1.82, 95% CI: 1.11–2.99) (Figure 1). A sensitivity analysis by removing one study [27] was conducted to evaluate the stability of the result. This analysis confirmed the stability of our result without apparent fluctuation (*I*^2^ = 0%, *P*=0.006, OR = 1.57, 95% CI: 1.14–2.15).

## 4. Discussion

The persistence of HBV infection in CHB is due to the reservoir of HBV covalently closed circular DNA (cccDNA) in the nucleus of infected hepatocytes, which serves as a template for viral transcription [30]. Antiviral therapies inhibit HBV DNA polymerase but do not directly target cccDNA, which indicates that the cccDNA remains present in the liver and is transcriptionally silent. HBV infection increases the levels of inflammatory and immunesuppressive cytokines. High levels of viremia would infect liver progenitor cells, which could be involved in HCC [31]. Our study made a threshold of HBV load at 2000 IU/ml, because if it is exceeded, the risk of disease progression and HCC will increase [32]. In general, the HBV-related end-stage liver disease (including LC and HCC) occurs decades after exposure [33]. Serum HBV DNA level is not only an effective predictor of the risk of LC and HCC [34, 35] but also a predictor of IFNs response in CHB patients [36]. It was reported that patients with deficiency syndrome including KYDS possessed a higher level of HBV DNA, most of which were in severe stage of CHB with splenomegaly [37] and diagnosed as level C by the Child-Pugh score [38]. In this research, HBV DNA level was also shown higher in KYDS patients than in non-KYDS patients. We speculate that CHB patients diagnosed as KYDS are easier to be progressed to LC or HCC than other TCM syndromes. However, a large sample size of patients should be used to verify the hypothesis.

Our study found a higher frequency of *HLA-DRB1∗08*:*03* amongst CHB patients compared to healthy controls. Considering that the small simple size would affect the reliability of the result, we performed a meta-analysis of the association between *HLA-DRB1∗08* and the susceptibility of CHB. The consequence of meta-analysis demonstrates the reliability of our result. *HLA-DRB1∗08*:*03* was also reported to be significantly associated with sufficient hepatitis B vaccine response [39]. The amino acid sequence encoded by *DRB1∗08*:*03* allele confers both susceptibility and progression in patients with primary biliary cirrhosis, in which the incidence of glycine-13, tyrosine-16, and leucine-74 was higher. Therefore, *HLA-DRB1∗08*:*03* might be a crucial locus associated with liver disease, especially for HBV susceptibility and efficacy.

There are few studies about the association of TCM syndrome with HLA polymorphism. In this study, *HLA-DQB1∗02*:*01* and *HLA-DRB1∗03*:*01* were proved to be susceptible genes of KYDS of CHB patients. *HLA-DQB1∗02*:*01* and *HLA-DRB1∗03*:*01* were conferred to have susceptible effect on chronic HBV infection [40, 41] and nonrespondence to HBV vaccination [42, 43]. *HLA-DQB1∗02*:*01* was reported to be significantly associated with familial aggregation and drug resistance in HBV infection [44]. The frequency of *HLA-DQB1∗02* is increased in HCC familial aggregation [45]. *HLA-DRB1∗03*:*01* associated with persistent infection of HBV [41]. Previous studies have found that KYDS possessed poor antiviral efficacy and was susceptible to drug resistance [46]; besides, it was in high risk of developing to HCC [47]. HBV hepatitis is largely noncytopathic, with host immune playing a critical role in liver injury and virus control [48]. Levels of host immune activation in response to HBV mediate liver injure and virus control, which could display elevated ALT activity and HBV DNA levels [49]. A study including 347,859 patients based on meta-analysis has found that HCC incidence of CHB increased substantially from the inactive carrier status to chronic hepatitis and compensated cirrhosis. The 5-year cumulative HCC risks were, respectively: 0.3%, 2.4%, and 15.5% in East Asia [33]. Patients with a family history of HCC multiplies the risk of HCC at each stage of infection [50] and HBV DNA serum loads >2000 IU/ml increases the HCC incidence rate [33]. Therefore, CHB patients with KYDS are in high risk of progressing to LC and HCC. The survival analysis need to be further researched to verify this conclusion.

In addition, Chinese medicine can significantly enhance the early response of NAs in the treatment of CHB [51] and reduce the side effects of NAs [52]. Clinic research and cell research have found that “Bushen” (therapeutic method based on KYDS in TCM) could improve the level of CD80, CD86, CD1a, and HLA-DR in HBV infection. Meanwhile, level of IL-10 could be reduced [53, 54]. It has been reported that “Bushen” prescription had effects on anti-inflammation and anti-HBV in HBV transgenic mice. The mechanism was found to be related to the MAVS-mediated signaling pathway [55]. Additionally, according to broad-range metabolomics coupled with network analysis, it was found that formulation-based TCM syndromes might prevent the pathological process of liver-kidney yin deficiency syndrome (LKYDS) through regulating the aging genes, such as CAV1 and ACO1 [56]. “Bushen” based on TCM theory could improve the immune system and delay the progression of the disease. All these findings indicate a significant role of KYDS in CHB. We should pay more attention to KYDS in CHB.

The current study also has several limitations. First, the small sample size and less research grouping would lead to result bias; however, the present outcomes could be regarded as an exploratory step to explore the objectivity of TCM syndromes. Second, we did not validate our results in independent samples. In the next study, we will enlarge the sample size and apply trans-ethnic examinations to verify the results.

## 5. Conclusions

In this study, we found an association between *HLA-DRB1* and *HLA-DQB1* polymorphisms and TCM syndromes. *HLA-DRB1∗0301* and *HLA-DQB1∗0201* are susceptible genes of KYDS. It helps to identify the biological mechanism of KYDS patients of CHB in high risk of progression to LC and HCC. These findings provide more opportunities for a deep understanding of TCM syndromes and exert TCM superiority in CHB treatment.

## Figures and Tables

**Table 1:** Subjects characteristics.

Variables	KYDS (*n* = 28)	Non-KYDS (*n* = 46)	HC (*n* = 31)	*P* value	OR (95% CI)
Age (*y*)^†^	37.43 ± 8.02	35.98 ± 9.75	37.48 ± 9.35	0.21	NA
Female (%)^‡^	7 (25.00%)	15 (32.61%)	11 (35.50%)	0.67	NA
HBeAg-positive, *n* (%)^‡^	11 (46.43%)	19 (47.83%)	NA	0.86	0.92 (0.35–2.40)
HBV DNA (≥2000 IU/ml, *n* (%)^‡^	17 (60.70%)	13 (28.30%)	NA	0.01^*∗*^	3.53 (1.32–9.46)
TBIL (*μ*mol/L)^§^	15.55 (9.68–21.23)	13.45 (11.18–17.85)	NA	0.62	NA
ALT (*μ*/L)^§^	51.55 (35.00–86.25)	34.50 (28.00–52.50)	NA	0.12	NA
AST (*μ*/L)^§^	41.50 (25.25–68.50)	39.00 (22.75, 79.25)	NA	0.49	NA

KYDS, kidney yang deficiency syndrome; HC, healthy control; TBIL, total bilirubin; ALT, alanine aminotransferase; AST, aspartate aminotransferase; NA, not available. ^†^Means ± SD. ^‡^Numbers and percentages. ^§^Median and interquartile range. ^*∗*^Significant *P* values (<0.05).

**Table 2:** Comparison of the frequencies of *HLA-DQB1*, *HLA-DRB1* alleles between CHB group and control group.

Allele	Frequencies in CHB Group (2*n* = 148)	Frequencies in control Group (2*n* = 62)	OR (95% CI)	*P* value
*DQB1* *∗* *02*:*01*	12 (8.11%)	3 (4.84%)	1.78 (0.47–6.80)	0.55
*DQB1* *∗* *02*:*02*	4 (2.70%)	3 (4.84%)	0.53 (0.11–2.54)	0.42
*DQB1* *∗* *03*:*01*	29 (19.59%)	19 (30.65%)	0.41 (0.17–0.96)	0.04^ *∗* ^
*DQB1* *∗* * 03*:*02*	6 (4.05%)	3 (4.84%)	0.82 (0.19–3.53)	0.72
*DQB1* *∗* *03*:*03*	35 (23.65%)	10 (14.52%)	2.19 (0.89–5.40)	0.08
*DQB1* *∗* 04:01	4 (2.70%)	0 (0.00%)	—	—
*DQB1* *∗* 05:01	3 (2.05%)	6 (9.68%)	0.46 (0.11–1.93)	0.38
*DQB1* *∗* *05*:*02*	18 (12.16%)	4 (6.45%)	2.17 (0.67–7.04)	0.20
*DQB1* *∗* *05*:*03*:*01G*	8 (5.41%)	5 (8.06%)	0.63 (0.19–2.11)	0.52
*DQB1* *∗* *06*:*01*	18 (12.16%)	4 (6.45%)	2.17 (0.67–7.04)	0.19
*DQB1* *∗* *06*:*07*	0 (0.00%)	1 (1.61%)	—	—
*DQB1* *∗* *06*:*10*	1 (0.01%)	0 (0.00%)	—	—
*DRB1* *∗* *04*:*01*	0 (0.00%)	1 (1.61%)	—	—
*DRB1* *∗* *04*:*05*	4 (2.70%)	0 (0.00%)	—	—
*DRB1* *∗* *04*:*06*	6 (4.05%)	2 (3.23%)	1.28 (0.24–6.72)	1
*DRB1* *∗* *12*:*01*	2 (1.35%)	5 (8.06%)	0.14 (0.03–0.79)	0.02^*∗*^
*DRB1* *∗* *12*:*02*	19 (12.84%)	7 (11.29*k*%)	1.18 (0.44–3.19)	0.81
*DRB1* *∗* *13*:*02*	0 (0.00%)	1 (1.61%)	—	—
*DRB1* *∗* *13*:*12*	2 (1.35%)	3 (4.84%)	0.26 (0.04–1.64)	0.15
*DRB1* *∗* *14*:*01*:*01G*	10 (6.76%)	5 (8.06%)	0.81 (0.25–2.61)	0.76
*DRB1* *∗* *14*:*04*	1 (0.68%)	1 (1.61%)	0.41 (0.03–6.79)	0.51
*DRB1* *∗* *14*:*05*	3 (2.03%)	2 (3.23%)	0.61 (0.10–3.86)	0.63
*DRB1* *∗* *15*:*01*	15 (10.14%)	5 (8.06%)	1.32 (0.44–4.02)	0.62
*DRB1* *∗* *15*:*02*	0 (0.00%)	2 (3.23%)	—	—
*DRB1* *∗* *01*:*01*	2 (1.35%)	1 (1.61%)	0.83 (0.07–9.54)	1
*DRB1* *∗* *03*:*01*	12 (8.11%)	3 (4.84%)	1.81 (0.47–6.91)	0.54
*DRB1* *∗* *07*:*01*	5 (3.38%)	3 (4.84%)	0.68 (0.15–3.02)	0.69
*DRB1* *∗* *08*:*03*	15 (10.14%)	1 (1.61%)	7.63 (0.96–60.53)	0.04^*∗*^
*DRB1* *∗* *09*:*01*	35 (23.65%)	10 (16.12%)	1.89 (0.78–4.55)	0.16
*DRB1* *∗* *10*:*01*	1 (0.68%)	0 (0.00%)	—	—
*DRB1* *∗* *11*:*01*	9 (6.08%)	4 (6.45%)	0.94 (0.27–3.30)	1

CHB, chronic hepatitis B. ^*∗*^significant *P* values (<0.05).

**Table 3:** Comparison of the frequencies of *HLA-DQB1*, *HLA-DRB1* alleles between KYDS group and non-KYDS group.

Allele	Frequencies in KYDS Group (2*n* = 56)	Frequencies in non-KYDS Group (2*n* = 92)	OR (95% CI)	*P* value
*DQB1* *∗* *02*:*01*	8 (14.29%)	4 (4.35%)	4.20 (1.13–15.61)	0.04^*∗*^
*DQB1* *∗* *02*:*02*	2 (3.57%)	2 (2.17%)	1.69 (0.23–12.75)	0.63
*DQB1* *∗* *03*:*01*:*01G*	13 (23.21%)	18 (19.57%)	1.35 (0.52–3.49)	0.55
*DQB1* *∗* *03*:*02*	2 (3.57%)	4 (4.35%)	0.81 (0.14–4.73)	1
*DQB1* *∗* *03*:*03*	13 (23.21%)	22 (23.91%)	0.57 (0.23–1.38)	0.21
*DQB1* *∗* *04*:*01*	1 (1.79%)	3 (3.26%)	0.53 (0.05–5.37)	1
*DQB1* *∗* *05*:*01*	2 (3.57%)	1 (1.09%)	3.46 (0.30–40.06)	0.55
*DQB1* *∗* *05*:*02*	5 (8.93%)	13 (14.13%)	0.55 (0.17–1.76)	0.31
*DQB1* *∗* *05*:*03*:*01G*	2 (3.57%)	6 (6.52%)	0.51 (0.10–2.74)	0.70
*DQB1* *∗* *06*:*07*	0 (0.00%)	0 (0.00%)	—	—
*DQB1* *∗* *06*:*10*	0 (0.00%)	1 (0.01%)	—	—
*DQB1* *∗* *06*:*01*	7 (0.13%)	11 (0.65%)	1.06 (0.35–3.16)	0.96
*DRB1* *∗* *04*:*01*	0 (0.00%)	0 (0.00%)	—	—
*DRB1* *∗* *04*:*05*	1 (1.79%)	3 (3.26%)	0.53 (0.05–5.37)	1
*DRB1* *∗* *04*:*06*	2 (3.57%)	4 (4.34%)	0.81 (0.14–4.73)	1
*DRB1* *∗* *12*:*01*:*01G*	2 (3.57%)	0 (0.00%)	—	—
*DRB1* *∗* *12*:*02*	8 (14.29%)	11 (11.96%)	1.35 (3.89–0.47)	0.58
*DRB1* *∗* *13*:*02*	0 (0.00%)	0 (0.00%)	—	—
*DRB1* *∗* *13*:*12*	1 (1.79%)	1 (1.09%)	1.67 (0.10–27.75)	1
*DRB1* *∗* *14*:*01*:*01G*	1 (1.79%)	9 (9.78%)	0.15 (0.02–1.28)	0.08
*DRB1* *∗* *14*:*04*	0 (0.00%)	1 (1.09%)	—	—
*DRB1* *∗* *14*:*05*	2 (3.57%)	1 (1.09%)	3.46 (0.30–40.06)	0.55
*DRB1* *∗* *15*:*01*	3 (5.35%)	12 (13.04%)	0.34 (0.09–1.33)	0.11
*DRB1* *∗* *15*:*02*	0 (0.00%)	0 (0.00%)	—	—
*DRB1* *∗* *01*:*01*	1 (1.79%)	1 (1.09%)	1.67 (0.10–27.75)	1
*DRB1* *∗* *03*:*01*	8 (14.29%)	4 (4.35%)	4.20 (1.13–15.61)	0.04^ *∗* ^
*DRB1* *∗* *07*:*01*	2 (3.57%)	3 (3.26%)	1.10 (0.17–7.04)	1
*DRB1* *∗* *08*:*03*	6 (10.71%)	9 (9.78%)	1.12 (0.35–3.58)	1
*DRB1* *∗* *09*:*01*	13 (23.21%)	22 (23.91%)	0.95 (0.37–2.42)	1
*DRB1* *∗* *10*:*01*	1 (1.79%)	0 (0.00%)	—	—
*DRB1* *∗* *11*:*01*	3 (5.36%)	6 (6.52%)	0.80 (0.18–3.49)	1
*DRB1* *∗* *16*:*02*	2 (3.57%)	5 (5.43%)	0.63 (0.11–3.49)	0.70

KYDS, kidney yang deficiency syndrome. ^*∗*^Significant *P* values (<0.05).

**Table 4:** Characteristics of the included studies in meta-analysis.

Study	Region	CHB (*n*)	Controls (*n*)	Type of control	HLA genotyping method	Reference
Fu	China	81	90	Healthy	PCR-SSOP	[24]
Han	China	72	200	Healthy	PCR-SSP	[16]
Jiang	China	52	106	Healthy	PCR-SSP	[25]
Kong	China	51	51	Healthy	PCR-SSP	[26]
Liu	China	102	201	Healthy	PCR-SBT	[27]
Yang	China	54	108	Healthy	PCR-SSP	[28]
Chen	China	120	400	Healthy	PCR-SSOP	[29]

PCR-SSOP, polymerase chain reaction with sequence-specific oligonucleotide probes; PCR-SSP, polymerase chain reaction with sequence-specific primers; PCR-SBT, polymerase chain reaction sequence-based typing.

## Data Availability

All data generated during this study are included in this published article and the supplementary information files.

## Abbreviations

CHB: Chronic hepatitis B
HBV: Hepatitis B virus
TCM: Traditional Chinese medicine
KYDS: Kidney yang deficiency syndrome
HLA: Human leukocyte antigen
PCR-SBT: Polymerase chain reaction sequence-based typing
LC: Liver cirrhosis
HCC: Hepatocellular carcinoma
NUCs: Nucleos(t)ide analogues
IFN: Interferon
ALT: Alanine aminotransferase
AST: Aspartate aminotransferase
TBIL: Total bilirubin.

## Supplementary Materials

Supplementary Materials Table S1: Diagnostic criteria of KYDS in TCM. Table S2: Inclusion and exclusion criteria. Table S3: Primer sequences. Table S4: Hardy–Weinberg equilibrium of HLA-DQB1 and HLA-DRB1.

## References

[1] Kao J.-H., Chen D.-S. (2002). Global control of hepatitis B virus infection. *The Lancet Infectious Diseases*.

[2] Organization WHO (2018). Hepatitis B.

[3] Chen D.-S., Locarnini S., Wait S. (2013). Report from a viral hepatitis policy forum on implementing the WHO framework for global action on viral hepatitis in North Asia. *Journal of Hepatology*.

[4] Seto W.-K., Lo Y.-R., Pawlotsky J.-M., Yuen M.-F. (2018). Chronic hepatitis B virus infection. *The Lancet*.

[5] Tsai T. Y., Livneh H., Hung T. H., Lin I -H., Lu M.-C., Yeh C.-C. (2017). Associations between prescribed Chinese herbal medicine and risk of hepatocellular carcinoma in patients with chronic hepatitis B: a nationwide population-based cohort study. *BMJ Open*.

[6] McCulloch M., Broffman M., Gao J., Colford J. M. (2002). Chinese herbal medicine and interferon in the treatment of chronic hepatitis B: a meta-analysis of randomized, controlled trials. *American Journal of Public Health*.

[7] Zhang L., Wang G., Hou W., Li P., Dulin A., Bonkovsky H. L. (2010). Contemporary clinical research of traditional Chinese medicines for chronic hepatitis B in China: an analytical review. *Hepatology*.

[8] Zeng X.-X., Bian Z.-X., Wu T.-X., Fu S.-F., Ziea E., Woon W. T. C. (2011). Traditional Chinese medicine syndrome distribution in chronic hepatitis B populations: a systematic review. *The American Journal of Chinese Medicine*.

[9] Xie H.-P., Yang H.-Z., Wu W.-K. (2014). Chinese medicine syndrome distribution of chronic hepatitis B virus carriers in immunotolerant phase. *Chinese Journal of Integrative Medicine*.

[10] Li C. (2017). Correlation between TCM syndromes and serum TGF-*β*1, HA, LN, IV-C and PC-III level of chronic hepatitis B after liver cirrhosis patients. *Journal of Chinese Medicine*.

[11] Lin B. H., Fang S. Q., Ye Y. (2002). Exploration on essence of spleen-kidney deficiency in middle-aged patients. *Chinese Journal of Integrated Traditional and Western Medicine*.

[12] Zhang Z. Y., Huang Y. S., Li W. B. (2018). The relationship between the syndrome type of Traditional Chinese Medicine and cellular immune function of chronic hepatitis B. *Chinese Journal of Integrated Traditional and Western Medicine on Liver Diseases*.

[13] Kamatani Y., Wattanapokayakit S., Ochi H. (2009). A genome-wide association study identifies variants in the HLA-DP locus associated with chronic hepatitis B in Asians. *Nature Genetics*.

[14] Nishida N., Sawai H., Matsuura K. (2012). Genome-wide association study confirming association of HLA-DP with protection against chronic hepatitis B and viral clearance in Japanese and Korean. *PloS One*.

[15] Hosaka T., Suzuki F., Kobayashi M. (2015). HLA-DPgenes polymorphisms associate with hepatitis B surface antigen kinetics and seroclearance during nucleot(s)ide analogue therapy. *Liver International*.

[16] Han Y.-N. (2005). Relationship of human leukocyte antigen class II genes with the susceptibility to hepatitis B virus infection and the response to interferon in HBV-infected patients. *World Journal of Gastroenterology*.

[17] Consortium M. S. (1999). Complete sequence and gene map of a human major histocompatibility complex. The MHC sequencing consortium. *Nature*.

[18] Yu F., Ma N., Zhang X. (2018). Comprehensive investigating of cytokine and receptor related genes variants in patients with chronic hepatitis B virus infection. *Cytokine*.

[19] Wang G. Q., Wang F. S., Cheng J. (2015). The guideline of prevention and treatment for chronic hepatitis B (2015 version). *Journal of Practical Hepatology*.

[20] Zhu W. F. (2002). *Diagnostics of Traditional Chinese Medicine*.

[21] Sayer D. C., Whidborne R., De Santis D., Rozemuller E. H., Christiansen F. T., Tilanus M. G. (2004). A multicenter international evaluation of single-tube amplification protocols for sequencing-based typing of HLA-DRB1 and HLA-DRB3,4,5. *Tissue Antigens*.

[22] Voorter C. E. M., Bruyn-Geraets D., Berg-Loonen E. M. (1997). High-resolution HLA typing for the DRB3/4/5 genes by sequence-based typing. *Tissue Antigens*.

[23] van Dijk A., Melchers R., Tilanus M., Rozemuller E. (2007). HLA-DQB1 sequencing-based typing updated. *Tissue Antigens*.

[24] Fu F., Chen Y., Liu X. X. (2006). A study on the association between chronic hepatitis B and HLA -DRB1. *Acta Academiae Medicine Xuzhou*.

[25] Jiang Y.-G., Wang Y. M., Liu T. H., Liu J. (2003). Association between HLA class II gene and susceptibility or resistance to chronic hepatitis B. *World Journal of Gastroenterology*.

[26] Kong X. J., Wang B. L. (2004). Study on the relationship between chronic hepatitis B and HLA-DRB1 allele. *Anhui Medecine Journal*.

[27] Liu Q., Tang Q., Yu W. (2017). Relevance analysis on HLA-DRB1 alleles and occult hepatitis B virus infection. *Chinese Journal of Blood Transfusion*.

[28] Yang G. T., Liu J., Xu D. Z. (2005). HLA-DRB1 genotyping and its relation with chronic hepatitis B patients of Han ethnic in Shanxi Area with HBV infection. *Medical Journal of Chinese People’s Liberation Army*.

[29] Han Y., Jiang Z. Y., Jiao L. X. (2012). Association of human leukocyte antigen-DRB1 alleles with chronic hepatitis B virus infection in the Han Chinese of Northeast China. *Molecular Medicine Reports*.

[30] Laras A., Koskinas J., Dimou E., Kostamena A., Hadziyannis S. J. (2006). Intrahepatic levels and replicative activity of covalently closed circular hepatitis B virus DNA in chronically infected patients. *Hepatology*.

[31] Dusséaux M., Masse-Ranson G., Darche S. (2017). Viral load affects the immune response to HBV in mice with humanized immune system and liver. *Gastroenterology*.

[32] Chen G., Lin W., Shen F., Iloeje U. H., London W. T., Evans A. A. (2006). Past HBV viral load as predictor of mortality and morbidity from HCC and chronic liver disease in a prospective study. *The American Journal of Gastroenterology*.

[33] Raffetti E., Fattovich G., Donato F. (2016). Incidence of hepatocellular carcinoma in untreated subjects with chronic hepatitis B: a systematic review and meta-analysis. *Liver International*.

[34] Chen C.-J. (2006). Risk of hepatocellular carcinoma across a biological gradient of serum hepatitis B virus DNA level. *Jama*.

[35] Iloeje U. H., Yang H. I., Su J., Jen C. L., You S. L., Chen C. J. (2006). Predicting cirrhosis risk based on the level of circulating hepatitis B viral load. *Gastroenterology*.

[36] van der Eijk A. A., Niesters H. G. M., Hansen B. E. (2006). Quantitative HBV DNA levels as an early predictor of nonresponse in chronic HBe-antigen positive hepatitis B patients treated with interferon-alpha. *Journal of Viral Hepatitis*.

[37] Lei C. G., Qin J. F., Cai L. (2017). Study on the correlation between TCM syndromes of chronic hepatitis B and clinical laboratory indicators. *Journal of Basic Chinese Medicine*.

[38] Li H. L. (2013). *To Investigate the Correlation between Deficiency Syndrome of Traditional Chinese Medicine and Main Indicators of Liver Function in Hepatitis B Cirrhosis of the Liver Decompensation Period*.

[39] Sakai A., Noguchi E., Fukushima T. (2017). Identification of amino acids in antigen-binding site of class II HLA proteins independently associated with hepatitis B vaccine response. *Vaccine*.

[40] Liu C., Cheng B. (2007). Association of polymorphisms of human leucocyte antigen-DQA1 and DQB1 alleles with chronic hepatitis B virus infection, liver cirrhosis and hepatocellular carcinoma in Chinese. *International Journal of Immunogenetics*.

[41] Thio C. L., Carrington M., Marti D. (1999). Class II HLA alleles and hepatitis B virus persistence in African Americans. *The Journal of Infectious Diseases*.

[42] Singh R. (2007). A comparative review of HLA associations with hepatitis B and C viral infections across global populations. *World Journal of Gastroenterology*.

[43] Li Z.-K., Nie J.-J., Li J., Zhuang H. (2013). The effect of HLA on immunological response to hepatitis B vaccine in healthy people: a meta-analysis. *Vaccine*.

[44] Xie D. S., Zhang Q. B., Huang J. F. (2010). Association between familial chronic HBV infection and HLA-II gene polymorphism. *Chinese Journal of Public Health*.

[45] Liang H. P., Lu T. T., Li Z. Z. (2017). The relationship between HLA-DQB1*∗*02, 03 alleles as well as IFN-*γ*, IL-4 and familial aggregation of hepatocellular carcinoma in Guangxi Zhuang and Yao people. *Genomics and Applied Biology*.

[46] Cheng H. Q., Huang C. H., Li C. L. (2014). Relationship between traditional Chinese medicine syndrome of chronic hepatitis B and anti-viral curative effect and drug resistance rate by lamivudine. *Chinese Jjournal of Integrated Traditional and Western Medicine on Liver Diseases*.

[47] Gao Y., Jiang Q., Zhou X. (2004). HBV infection and familial aggregation of liver cancer: an analysis of case-control family study. *Cancer Causes & Control*.

[48] Rehermann B. (2013). Pathogenesis of chronic viral hepatitis: differential roles of T cells and NK cells. *Nature Medicine*.

[49] Hoofnagle J. H., Doo E., Liang T. J., Fleischer R., Lok A. S. F. (2007). Management of hepatitis B: summary of a clinical research workshop. *Hepatology*.

[50] Loomba R., Liu J., Yang H. I. (2013). Synergistic effects of family history of hepatocellular carcinoma and hepatitis B virus infection on risk for incident hepatocellular carcinoma. *Clinical Gastroenterology and Hepatology*.

[51] Liu Y. H., Zhang Y. H., Guo Y. (2008). Effect of synergy and influence on dendritic cell function that determining the treatment based on differentiation of symptoms and signs to chronic hepatitis B treated by lamivudine. *Chinese Archives of Traditional Chinese Medicine*.

[52] Hu X. Y., Zhang Y., Liu G. W., Nie H. M., Fan X. J., Zhong S. (2012). Influences of warming kidney prescription on antiviral therapeutic efficacy and creatine kinase in telbivudine-treated HBeAg-positive chronic hepatitis B patients. *Chinese Journal of Hepatology*.

[53] Ou S., Sun K. W., Peng J. P., Qi S. L., Wen J., Hu L. (2013). Effects of bushen Jiedu recipe and jianpi jiedu recipe containing plasma on dendritic cell of chronic hepatitis B virus infection patients under different immune states. *Chinese Journal of Integrated Traditional and Western Medicine*.

[54] Feng X. X., Wang L., Xing L. J. (2010). The function of dendritic cell in chronic hepatitis B patients with deficiency of kidney base on traditional Chinese medicine syndrome differentiation intervention. *Journal of Clinical Hepatology*.

[55] Zhang J. H., Zhen C., Sun X. H. (2019). Anti-inflammation antiviral effects and mechanisms of Bushen recipe based on MAVS mediated signal pathway. *Chinese Journal of Integrated Traditional and Western Medicine on Liver Diseases*.

[56] Zhai Y., Xu J., Feng L. (2019). Broad range metabolomics coupled with network analysis for explaining possible mechanisms of Er-Zhi-Wan in treating liver-kidney Yin deficiency syndrome of Traditional Chinese medicine. *Journal of Ethnopharmacology*.

