# Supplementary material for: HLA-DQB1/DRB1 Alleles Associate with Traditional Chinese Medicine Syndrome of Chronic Hepatitis B: A Potential Predictor of Progression
Source: Biomed Res Int. 2019 Nov 21;2019:8146937. doi: 10.1155/2019/8146937 (PMC6906876; doi:10.1155/2019/8146937)
Supplement: Supplementary Materials — Table S1: Diagnostic criteria of KYDS in TCM. Table S2: Inclusion and exclusion criteria. Table S3: Primer sequences. Table S4: Hardy–Weinberg equilibrium of HLA-DQB1 and HLA-DRB1. [file 8146937.f1.pdf]

## Table S1. Diagnostic criteria of KYDS in TCM

| | **Symptomes** |
|---|---|
| **Presenting symptoms** | a. soreness and weakness of waist and knees<br>b. chilly, cold limbs, and lacking in strength<br>c. hypaphrodisia |
| **Minor symptoms** | a. pale or dull complesxion<br>b. urine frequency or urgency or difficulty, or excessive at night<br>c. loose stool or dawn diarrhea<br>d. male impotentia, prospermia, or sterility. Female dysgenesis, leucorrhea increases, or amenia<br>e. with lightly purple tongue and smooth fur<br>d. with deep thready pulse, especially in the part of chi |
| **Criterion of differentiation of KYDS** | satisfied with two of the presenting symptoms(the ***a*** is necessary), or content with the ***a*** of the presenting symptom and two or more of the minor symptoms. |

## Table S2. Inclusion and exclusion criteria

| | |
|---|---|
| **Inclusion criteria** | a. satisfied with the diagnostic criteria of CHB |
| | b. aged between 18-45 years old |
| | c. provided written informed consent for participation |
| **Exclusion criteria** | a. patients diagnosed with liver cirrhosis, or cancer, or hepatic failure |
| | b. complicated with Drug-Induced Liver Injury, autoimmune liver disease, alcohol liver disease |
| | c. patients with mental illness or inability to cooperate |

## Table S3. Inclusion and exclusion criteria of meta-analysis

| | |
|---|---|
| **Inclusion criteria** | a. an independent case control study |
| | b. relevant genotype frequencies, or odds ratio(OR) and 95% confidence interval(CI) should be reported |
| | c. a full text study so that detail information could be acquired |
| **Exclusion criteria** | a. the control group was not healthy |
| | b. duplicate publication |
| | c. China wasn't the study region |
| | d. materials and methods were not well-described and reliable |

**Table S4. Hardy-Weinberg Equilibrium of *HLA-DQB1, HLA-DRB1***

| | KYDS | | | | NKYDS | | | | HC | | | |
|---|---|---|---|---|---|---|---|---|---|---|---|---|
| Locus | N[a] | $H_{obs}$[b] | $H_{exp}$[c] | P[d] | N[a] | $H_{obs}$[b] | $H_{exp}$[c] | P[d] | N[a] | $H_{obs}$[b] | $H_{exp}$[c] | P[d] |
| *HLA-DQB1* | 28 | 0.8571 | 0.8578 | 0.54 | 46 | 0.8478 | 0.8629 | 0.95 | 31 | 0.9355 | 0.8509 | 0.83 |
| *HLA-DRB1* | 28 | 0.8929 | 0.8961 | 0.44 | 46 | 0.8696 | 0.8884 | 0.88 | 31 | 0.9355 | 0.9344 | 0.49 |

N[a], Sample number; $H_{obs}$[b], Observed heterozygosity; $H_{exp}$[c], Expected heterozygosity; P[d], *P* value
